# A cross-country database of COVID-19 testing

**DOI:** 10.1038/s41597-020-00688-8

**Published:** 2020-10-08

**Authors:** Joe Hasell, Edouard Mathieu, Diana Beltekian, Bobbie Macdonald, Charlie Giattino, Esteban Ortiz-Ospina, Max Roser, Hannah Ritchie

**Affiliations:** 1 Oxford Martin Programme on Global Development, University of Oxford, Oxford, United Kingdom; 2 Our World in Data, University of Oxford, Oxford, United Kingdom; 3 Department of Social Policy and Intervention, University of Oxford, Oxford, United Kingdom; 4 School of Economics, University of Nottingham, Nottingham, United Kingdom; 5 Department of Political Science, Stanford University, Stanford, United States

**Keywords:** Infectious diseases, Epidemiology

## Abstract

Our understanding of the evolution of the COVID-19 pandemic is built upon data concerning confirmed cases and deaths. This data, however, can only be meaningfully interpreted alongside an accurate understanding of the extent of virus testing in different countries. This new database brings together official data on the extent of PCR testing over time for 94 countries. We provide a time series for the daily number of tests performed, or people tested, together with metadata describing data quality and comparability issues needed for the interpretation of the time series. The database is updated regularly through a combination of automated scraping and manual collection and verification, and is entirely replicable, with sources provided for each observation. In providing accessible cross-country data on testing output, it aims to facilitate the incorporation of this crucial information into epidemiological studies, as well as track a key component of countries’ responses to COVID-19.

## Abstract (summary)

**Table:** 

Measurement(s)	Test • Extent
Technology Type(s)	digital curation
Factor Type(s)	country • temporal interval
Sample Characteristic - Organism	Coronaviridae
Sample Characteristic - Location	global

Machine-accessible metadata file describing the reported data: 10.6084/m9.figshare.12924755

## Background & Summary

Across the world, researchers and policymakers look to confirmed counts of cases and deaths to understand and compare the spread of the COVID-19 pandemic. However, data on cases and deaths can only be meaningfully interpreted alongside an accurate understanding of the extent and allocation of virus testing^1^. Two countries reporting similar numbers of confirmed cases may in fact have very different underlying outbreaks: other things being equal, a country that tests less extensively will find fewer cases.

Many countries now publish official COVID-19 testing statistics, but the insights offered by these numbers remain relatively unexplored both in public discourse and scientific research. This may be because of barriers limiting access to this data: the statistics are scattered across many websites and policy documents, in a range of different formats. No international authority has taken on the responsibility for collecting and reporting testing data. We developed a new global database to address this lack of access to reliable testing data, thereby complementing the available international datasets on death and case counts^2^.

The database consists of official data on the number of COVID-19 diagnostic tests performed over time across 94 countries (as of 31 August 2020). We rely on figures published in official sources, including press releases, government websites, dedicated dashboards, and social media accounts of national authorities. We do not include in our database figures that explicitly relate to only partial geographic coverage of a country (such as a particular region or city).

The resulting database is (i) updated regularly through a combination of automated scraping and manual collection and verification, and (ii) entirely replicable, with sources provided for each observation.

In addition, the database includes extensive metadata providing detailed descriptions of the data collected for each country. Such information is essential due to heterogeneity in reporting practices, most notably regarding the units of measurement (people tested, cases tested, tests performed, samples tested, etc). Series also vary in terms of whether tests pending results are included, the time period covered, and the extent to which figures are affected by aggregation across laboratories (private and public) and subnational regions.

**Fig. 1:**
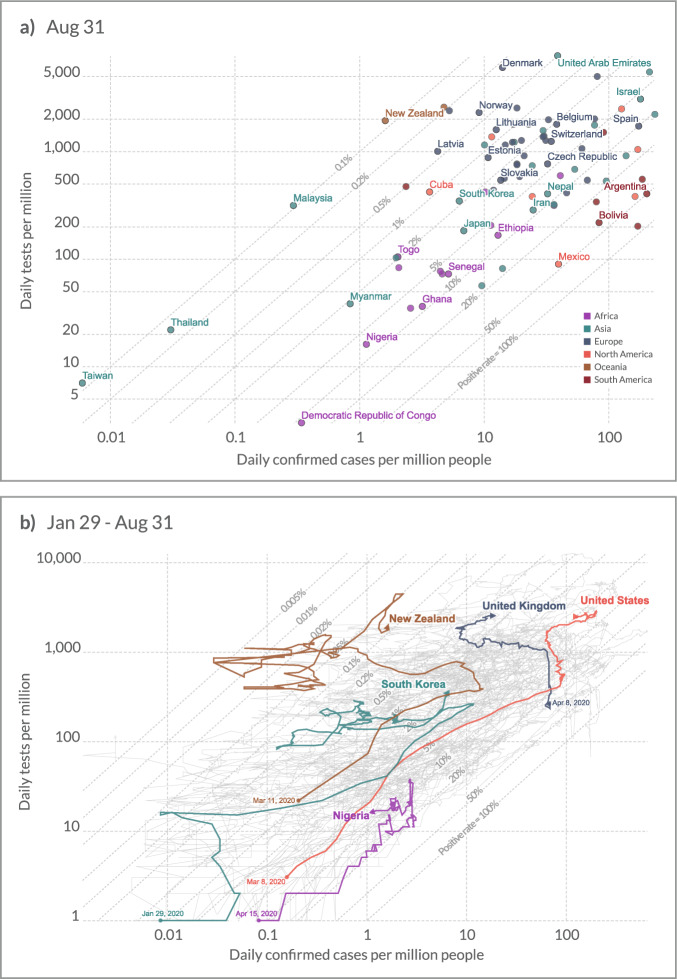
COVID-19: Daily tests vs. Daily new confirmed cases, per million Panel A plots the number of daily tests against the number of daily confirmed cases per million of the population, as of August 31. Both are given as the rolling 7-day average. Panel B shows the same two variables over time for five selected countries. All axes are given on a log scale. Note that comparisons of testing data between countries are affected by reporting differences. Details for each country can be found in the metadata. Data on confirmed cases is from the European CDC.

The comprehensiveness of our database enables comparisons of the extent of testing between countries and over time — in absolute terms, but also relative to countries’ population, and to death or confirmed case counts (Fig. 1).

Such variation offers crucial insights into the pandemic. At the most basic level, it is clear that a country that tests very few people — such as the Democratic Republic of Congo, or Nigeria (Fig. 1a) — can only have very few confirmed cases. The number of performed tests should be seen as an upper limit for the number of confirmed cases.

Further, high positive test rates (Fig. 1 — see reference lines) may help identify severe underreporting of cases. The relationship between test positivity rate and case underreporting has been explored in the context of other infectious diseases^3^. In terms of COVID-19, this link is discussed by Ashish Jha and colleagues at the Harvard Global Health Institute, who provide a sketch of the relationship between cases, deaths and the positivity rate in the United States (see https://globalepidemics.org/2020/04/18/why-we-need-500000-tests-per-day-to-open-the-economy-and-stay-open). In a more formal analysis, Golding *et al*.^4^ find that their modelling estimates of the case ascertainment rate are weakly correlated (Kendall’s correlation coefficient of 0.16) with the number of tests per case — the inverse of the test positivity rate — derived from our database, with a positive relationship evident in the range of 10–35 tests per case^4^. The Institute for Health Metrics and Evaluation (IHME) include testing data sourced from our database in their COVID-19 models (www.healthdata.org/covid/faqs#differences%20in%20modeling). In bringing this data together, our hope is that we will facilitate future research in this direction.

More generally, our aim is to provide an essential complement to counts of confirmed cases and deaths. These are the figures that guide public policy, both in the initiation of control measures and as they start to be relaxed. But without the context provided by data on testing, reported cases and deaths may offer a very distorted view on the true scale and spread of the COVID-19 pandemic.

## Methods

The database consists of two parts, provided for each included country: (1) a time series for the cumulative and daily number of tests performed, or people tested, plus derived variables (discussed below); (2) metadata including a detailed description of the source and any available information on data quality or comparability issues needed for the interpretation of the time series.

For most countries, a single time series is provided: either for the number of people tested, or the number of tests performed. For a few countries for which both are made available, both series are provided. In such cases, metadata is provided for each separate series.

### Data collection methods

The time series data is collected by a combination of manual and automated means. The collection process differs by country and can be categorized into three broad categories.

Firstly, for a number of countries, figures reported in official sources — including press releases, government websites, dedicated dashboards, and social media accounts of national authorities — are recorded manually as they are released.

Secondly, where such publications are released in a regular, machine-readable format, or where structured data is published at a stable location, we have automated the data collection via R and Python scripts that we execute every day. These are regularly audited for technical bugs by checking their output against the original official sources (see ‘Technical Validation’, below).

Lastly, in some instances where manual collection has proven prohibitively difficult, we source data from non-official groups collecting the official data, most often on GitHub. These are also regularly audited for accuracy against the original official sources (see ‘Technical Validation’, below).

Any available information on data quality or comparability issues needed for the interpretation of the time series is gathered and summarized manually into detailed metadata for each series, guided by a checklist of data quality questions (see Data Records, below).

### Criteria for inclusion

Throughout the pandemic, PCR tests for the presence of SARS-CoV-2 RNA have in general been the basis for COVID-19 case confirmation, in line with WHO recommendations^5^. Since the primary purpose of the database is to provide information on testing volumes specifically to aid the interpretation of data on confirmed cases, it is exclusively this category of testing technologies that the database aims to include.

In order to be included, a data point for a given country must report an aggregate figure that includes both negative tests (or negatively tested individuals) plus positive tests (or confirmed cases). The units (whether the number of *tests* or *individuals* is being counted) must be consistent across positive and negative outcomes.

The aggregate figure must refer to a known time period — for instance, the number of tests performed in the last day or week. However, where a cumulative total is provided, it is not a requirement that the specific start date to which the cumulative count relates must be specified, provided that it is clear that the figure aims to capture the whole of the relevant outbreak period.

Figures relating to testing ‘capacity’ or to rough indications of average testing output, or to the number of tests that have been distributed (rather than actually performed) are not included in the database.

Where figures for pending tests are provided separately by a source these are excluded from our counts. Where they cannot be separated, the figures including pending tests are reported. Details concerning pending tests for individual countries can be found in the metadata.

### Raw data and derived variables

The database provides a time series for both the cumulative number of tests (or people tested) and for daily new tests. Exactly how these series are derived depends on the way the raw data is reported by the source.

Where a source provides a complete time series for daily tests, we derive an additional cumulative series as the simple running total of the raw daily data. Where a source provides cumulative figures, we derive an additional daily series as the day-to-day change observed in consecutive observations.

**Fig. 2:**
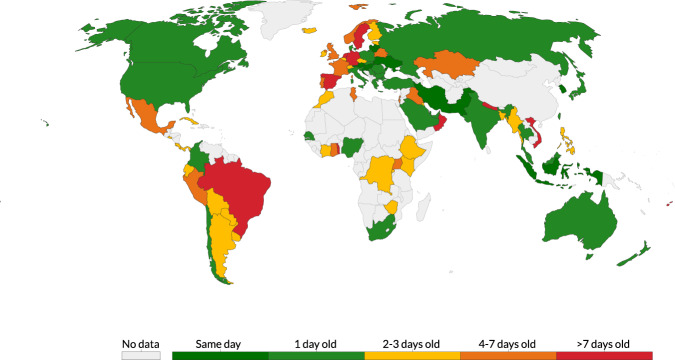
Number of days between the most recent observation for each country and the date of updating (31 August 2020). Because countries update their testing data at different frequencies, the latest data in the database for each country does not in all cases refer to the date the database was updated.

In many cases the source data is not available at a daily frequency (Fig. 2). In order to facilitate cross-country comparisons over time, we derive an additional ‘smoothed’ daily testing series calculated as the seven-day moving average over a complete, linearly interpolated daily series (described in more detail in the Data Records section below).

### Retrospective revisions in the source data

Due to the efforts to produce timely data, official testing figures are subject to frequent retrospective revisions. This can occur for instance where some laboratories have longer reporting delays than others, and previously uncounted tests are then subsequently included.

This issue presents no difficulties where sources provide an updated time series within which such revisions are appropriately incorporated; for instance, by backdating the additional tests to the date they were performed.

However, a number of the sources we rely on provide only a ‘snapshot’ of the current cumulative figure, with no time series. We construct our cumulative and daily testing time series from the sequence of these ‘snapshots’. For these cases, retrospective revisions do impact our data since revisions to the data are included on the day the revision is made, not when the revised tests occurred. Typically, this results in only small deviations in the cumulative figure in proportional terms, but the derived daily testing series can be impacted more meaningfully. At the extreme, in a few cases, such revisions result in a fall in the cumulative total from one day to the next, implying a negative number of tests for that day.

This issue is mitigated in two ways. Firstly, given that much of retrospective revision relates to testing conducted over the last few days, the ‘smoothed’ daily time series we derive reduces some of the artificial volatility introduced. Secondly, we alert the user as to which data is subject to such concerns as part of the information included in the metadata (see below).

## Data Records

A copy of the database has been uploaded to figshare^6^. This provides a version of the database as it stood at the time of submission, on 31 August 2020.

A live version of the database, which continues to be updated, can be downloaded from a public GitHub repository (https://github.com/owid/covid-19-data/tree/master/public/data/testing) in CSV, XLSX, and JSON formats, which may be imported into a variety of software programs.

### Structure

The database consists of two components: a time series file including observations of cumulative and daily testing (*covid-testing-all-observations.csv*), and metadata (*covid-testing-source-details.csv*). Each row in the metadata table provides source details (discussed below) corresponding to a given country-series (i.e. the combination of *Country* and *Series* fields make up a unique ID within *covid-testing-source-details.csv*). The time series for cumulative and daily testing for each country-series is then provided in the *covid-testing-all-observations.csv* file.

In addition, we provide the raw data (*raw-collected-data.csv*), as collected from the source, in order to make it plain how our time series data is constructed from the original observations. We also provide the United Nations population data for 2020 (*un-2020-population.csv*) used to derive the per capita measures included in the time series.

### Description of fields

#### Common to covid-testing-source-details.csv, covid-testing-all-observations.csv, and raw-collected-data.csv

##### Country

Each observation relates to testing conducted within the indicated country. We do not include in our database figures that explicitly relate to only partial geographic coverage of a country (such as a particular region or city). The country’s 3-letter ISO 3166-1 code is also provided as a separate field.

##### Units

A short description of the unit of observation of the collected testing figures, selected out of three possible categories: “people tested”, “tests performed”, “samples tested”. Series for which it was not possible to discern the category are labelled as “units unclear”.

##### Series

Multiple series (e.g. people tested and samples tested) are included for some countries, and are demarcated by this field.

#### Common to covid-testing-all-observations.csv and raw-collected-data.csv

##### Date

Depending on the source, this may relate to the date on which samples were taken, analyzed, or registered, or simply the date they were included in official figures (see ‘Retrospective revisions in the source data’, above). In general, sources try to provide testing data relating to a given, stable cut-off time each day. Where significant changes in reporting windows have been found, these have been noted in the *Notes* field (see below).

##### Cumulative total

The reported cumulative amount of testing as of *Date*. The specific date to which the cumulative figures date back to, if known, is provided in the metadata (see below). In many cases this is not explicitly stated by a source, but only figures that appear to intend to capture the entire period of the testing response to COVID-19 outbreak within the country are included in the database. In *covid-testing-all-observations.csv*, for those sources only providing daily testing figures, this field is derived as the running total of the raw daily data, and is also provided per thousand people of the country’s 2020 population.

##### Daily change in cumulative total

Broadly, this field may be interpreted as the number of new tests (or people tested) per day. For sources that report new tests per day directly, this field in *covid-testing-all-observations.csv* is identical to the raw data presented in *raw-collected-data.csv*. For sources that report only cumulative testing figures, the field is derived as the day-to-day change observed in consecutive observations of the raw *Cumulative total* data. This may fail to correspond to the true number of new tests for that date where the source has included retrospective revisions in the cumulative totals (see ‘Retrospective revisions in the source data’, above). In *covid-testing-all-observations.csv*, this series is also provided per thousand people of the country’s 2020 population.

##### Source URL

A URL at which the specific observation of the corresponding raw data can be found.

##### Source label

The name of the source for the observation.

##### Notes

Contains any notes to aid the interpretation of this specific observation (above and beyond details that apply to the whole series, which are provided in *covid-testing-source-details.csv*).

#### Specific to *covid-testing-all-observations.csv*

##### 7-day smoothed daily change

As an outbreak progresses, flows of new tests per day, rather than cumulative figures, become more relevant for understanding trends. Daily testing figures however suffer from volatility created by reporting cycles. Moreover, since many sources do not provide data at daily intervals, figures for new tests per day are available with more limited coverage. To aid the cross-country analysis of testing volumes over time, we provide this short-term measure of testing output that aims to mitigate these two problems. It is calculated as the right-aligned rolling seven-day average of a complete series of daily changes. For countries for which no complete series of daily changes is available because of the reporting frequency of our source, we derive it by linearly interpolating the daily cumulative totals not available in the raw data, up to a maximum interval of 21 days. The exact code used to derive the 7-day smoothed daily change is available online (see ‘Code Availability’, below).

#### Specific to *covid-testing-source-details.csv*

##### Number of observations

The number of days for which raw observations are available.

##### Detailed description

A written summary of available information concerning the nature and quality of the source data needed for proper interpretation and cross-country comparison. The collation of this information is guided by a ‘checklist’ of data quality questions regarding: the unit of observation; which testing technologies figures relate to; whether tests pending results are included; the time period covered; and the extent to which figures are affected by aggregation across laboratories (private and public) and subnational regions. In practice the documentation we are able to provide is limited by that made available by the official source. We aim to include any information provided by the original source needed for the interpretation and comparison with other countries.

### Coverage

The database includes observation for 94 countries, covering 69% of the world’s population. Because of differences in the frequency at which countries publish testing data, coverage is somewhat lower for more recent periods: 62% of the world’s population is covered with figures relating to 30–31 August 2020; 45% is covered with figures relating to 24–31 August 2020 (Fig. 2).

## Technical Validation

The database represents a collation of publicly available data published by official sources. As such, the key quality concern for the database itself is whether it represents an accurate record of the official data. We employ four main strategies for ensuring this.

Firstly, all automated collection of data, whether obtained from official channels or from third-party repositories of official data, is subject to initial manual verification when it is added to our database for the first time.

Secondly, we employ a range of data validation processes, both for our manual and automated time series. We continually check for invalid figures such as negative daily test figures, out-of-sequence dates, or test positivity rates above 100% (by comparing testing data to confirmed case data), and we monitor each country for abrupt changes in daily testing rates.

Abrupt positive or negative daily changes are sometimes the result of data corrections in the official data, in which case our database includes them without alteration. These changes can be due, for example, to the deduplication of double-counted tests, or the addition of testing data that was previously not captured by the national system (see Table 1).

**Table 1:** Examples of abrupt daily changes in the testing database due to data corrections performed by the official source.

Country	Changes observed in the source data as of 31 August 2020
Ecuador	4,803 people added on 3 May 2020 3,743 people subtracted on 5 May 2020 1,377 people subtracted on 11 May 2020
Fiji	357 tests subtracted on 10 July 2020
Greece	53,889 samples added on 29 July 2020
Japan	453 people subtracted on 19 March 2020 909 people subtracted on 25 March 2020 2,262 people subtracted on 15 May 2020 48,382 people added on 18 June 2020
Malaysia	126,964 people added on 15 May 2020
Nigeria	8,760 samples added on 29 May 2020 29,602 samples added on 22 July 2020
Peru	25 people subtracted on 21 March 2020
Philippines	364 people subtracted on 8 June 2020
Poland	236,927 samples subtracted on 8 August 2020
Zimbabwe	5,055 tests added on 24 July 2020

In order to mitigate against large impacts due to reporting lags, we automatically exclude the most recent observation for a country if its daily number of new tests is less than half that of the previous observation. This is only applied to the most recent day in each time series: as soon as data for subsequent days becomes available, the data point is reinstated if the sharp fall is still present.

Thirdly, to monitor the ongoing reliability of third-party repositories of official data, we apply a continuous audit process, which will remain active as long as this dataset is updated. Each day, three observations are randomly drawn out of all observations in the database that have been obtained via third-party sources. For each selected observation, the recorded figure is manually checked against the direct official channel from which the repository purports to obtain the data. The sampling rate means that each third-party source we make use of is checked around once a week. Given that any discrepancies with official channels are likely to be clustered within particular sources, this provides a high degree of quality control on these sources on a timely basis. Where any discrepancies are noticed, we switch sources (for the entire time series) to either a different repository or to manual data collection directly from the official channel.

Finally, the testing data included in the database is viewed by tens of thousands of people every day, including many health researchers, policymakers and journalists, from which we receive a large amount of feedback concerning the data. This serves as a final, ‘crowd-sourced’ method of verification that has proven very effective, enabling any discrepancies between our data and that published in official channels to be flagged and resolved quickly.

## Data Availability

Code used for the creation of this database is not included in the files uploaded to figshare. Our scripts for data collection, processing, and transformation, are available for inspection in the public GitHub repository that hosts our data (https://github.com/owid/covid-19-data/tree/master/scripts/scripts/testing).
